# Identification of four novel variants in the *CDH23* gene from four affected families with hearing loss

**DOI:** 10.3389/fgene.2022.1027396

**Published:** 2022-11-17

**Authors:** Baoling Kang, Xinshu Lu, Jianjun Xiong, Yuan Li, Jinwen Zhu, Tao Cai

**Affiliations:** ^1^ Beijing Angel Gene Medical Technology Co., Ltd., Beijing, China; ^2^ College of Basic Medical Science, Jiujiang University, Jiujiang, China; ^3^ China-Japan Friendship Hospital, Beijing, China

**Keywords:** hearing loss, Cdh23, autosomal recessive, mutation, exome sequencing

## Abstract

**Background:** Hearing loss (HL) is the most common form of sensory disorder in humans. Molecular diagnosis of HL is important for genetic counseling for the affected individuals and their families.

**Methods:** To identify potential genetic causes, we performed whole-exome sequencing and related biomedical informatics for 351 non-syndromic HL patients and their family members.

**Results:** In the present study, we report the identification of four compound heterozygous variants in the *CDH23* gene from four affected families, including four novel variants (c.995C>A, p.T332K; c.2159G>A, p.R720Q; c.5534A>G, p.N1845S, and c.7055-1G>C) and two frequently reported variants (c.719C>T, p.P240L and c.4762C>T, p.R1588W).

**Conclusion:** Our findings significantly expanded the mutation spectrum of *CDH23*-associated autosomal recessive hearing loss.

## Introduction

Hearing loss (HL) is the most common form of sensory disorder in humans, which can have a profound impact on quality of life. Approximately 466 million people worldwide suffer from hearing problems, which is projected to reach 900 million by 2050 (Li et al., 2021a). In developed countries, approximately 80% of congenital hearing loss is genetic, most often autosomal recessive and nonsyndromic (Shearer et al., 1993). Genetic-related hearing loss can be subdivided into syndromic or nonsyndromic, based on the presence or absence of coinherited anomalies. To date, more than 150 genes have been found to cause hearing loss (Raviv et al., 2010). Approximately 60%–70% of cases of inherited nonsyndromic deafness are associated with autosomal recessive mutations while 20%–30% are autosomal dominant (Rehman et al., 2017).

Mutations in the *CDH23* gene were first identified in patients with Usher Syndrome (Bolz et al., 2001; Bork et al., 2001), which is a syndromic hearing loss (OMIM: 601067) The encoded protein cadherin 23 (CDH23) belongs to the cadherin superfamily, which constitutes a family of transmembrane proteins that mediate calcium-dependent cell-cell adhesion (Ramzan et al., 2020). To date, at least 492 different mutations in *CDH23* have been curated in Human Gene Mutation Database (HGMD). Among them, 40 different recessive mutations of *CDH23* are associated with non-syndromic deafness 12 (DFNB12, Phenotype ID 434809557). Most of the *CDH23* variants are associated with Usher syndrome type 1 or syndromic deafness (USH1D).

In the past 3 years, we recruited a cohort of 351 affected individuals with congenital sensorineural deafness using whole-exome sequencing and associated bioinformatics analysis. Here, we reported the identification of four novel variants in the *CDH23* gene from four probands with profound congenital sensorineural deafness.

## Materials and methods

### Affected individuals

A total of 351 affected individuals with congenital hearing loss and their parents in 323 families were recruited by local hospitals for exome sequencing analysis. Detailed medical histories were collected for all affected individuals and their families. A series of tests were performed for each of the affected individuals, such as high-resolution CT and magnetic resonance imaging (MRI) scans. All the participating individuals signed written informed consent.

### Exome sequencing and bioinformatic analyses

Blood samples from the affected individuals and their family members were taken for DNA extraction. Exome-enriched genomic libraries were prepared using the Agilent SureSelect Human Expanded All Exon V6 kit and sequenced on an Illumina NovaSeq6000 with an average of 100x coverage. Genomic reads were aligned for SNP calling and further analysis for identification of candidate causal variants which are predicted by multiple programs, such as Varsome, MutationTaster, Polyphen-2 and SIFT. Detected variants with MAF >0.001 based on gnomAD or in-house Chinese Exome Database were eliminated as previously described (Li et al., 2021b; Cai et al., 2022; Luo et al., 2022). Selected variants were further confirmed by Sanger sequencing with specific primers (Supplementary Table S1). Based on the wildtype 3-dimentional CDH23 protein model (https://swissmodel.expasy.org/), potential effects of the identified variants on CDH23 protein functions were predicted using SPDBV 4.10 software (https://swiss-pdb-viewer.software.informer.com/4.1/).

## Results

### Clinical findings of five affected individuals

**FIGURE 1:**
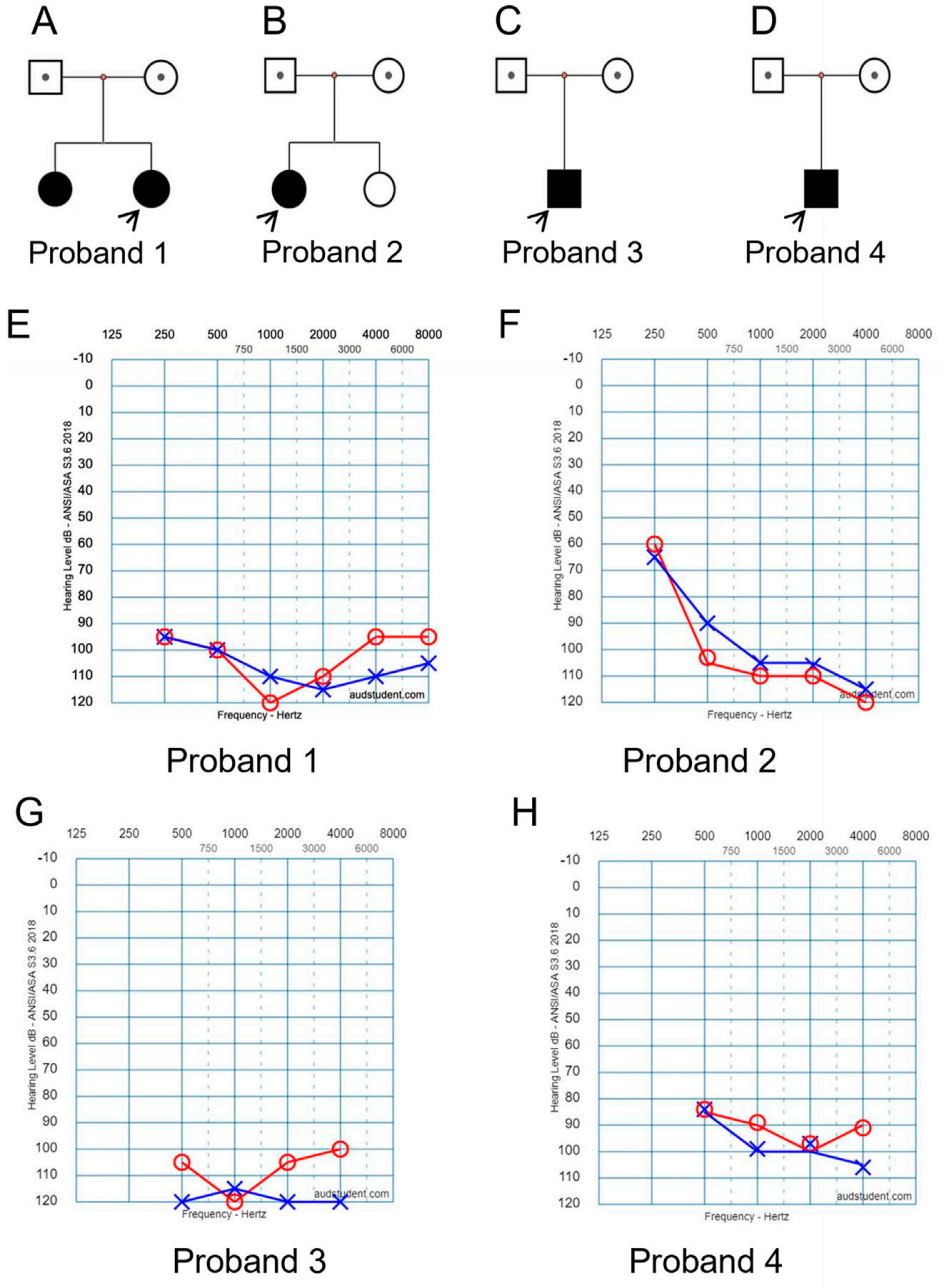
Clinical findings of affected four families. **(A–D)** Family trees. Probands are indicated by arrows. **(E–H)** Pure tone audiometry examinations show bilateral severe sensorineural hearing loss for the affected probands 1 to 4, respectively.

In family 1, two daughters were affected with hearing loss (Figure 1A). The proband was a 4 years old girl, who had normal motor development after birth, normal fine movements. Both parents had no family history of any genetic disorders.

In family 2, the proband was a 4 years old girl (Figure 1B). She did not pass newborn hearing screening. She could call “mom” when she was 7-month-old, and call “dad” around 9-month-old. She received a hearing aid at the age of 2.5 years and had a cochlear implant at the age of four. No family history of genetic disorders was found.

In family 3, the affected boy (Figure 1C) passed newborn hearing screening. He could call mom and dad around 1 year old. At 2-year and 3-month of age, he was diagnosed with bilateral profound/severe hearing loss. No other abnormalities or family history of genetic disorders were found.

In family 4, the affected boy (Figure 1D) was diagnosed with severe hearing loss when he was about 1 year old. No other abnormalities were found by physical examination. No family history of genetic disorders was found.

Hearing threshold tests using pure tone audiometry examinations show severe bilateral sensorineural hearing loss for all the affected probands (Figures 1E–H, respectively). The hearing threshold levels [Rt/Lt (dB)] for each of the probands are between 90/110 as listed in Table 1.

**TABLE 1:** Evaluation of seven different *CDH23* mutations identified in five patients with hearing loss.

Family	Threshold Rt/Lt (dB)	Allele 1	Varsome/ACMG	Allele 2	Varsome/ACMG
1 (2 affected)	100/100	c.719C>T; g.73330641C>T (p. Pro240Leu)	pathogenic; score: 14; ACMG: Pathogenic	c.5534A>G g.73544679A>G (p. Asn1845Ser)	Pathogenicity score: 13; ACMG: Likely Pathogenic
2 (4 years-old)	110/110 Profound	c.719C>T g.73330641C>T (p. Pro240Leu)	Pathogenicity Scores: 13; ACMG: Pathogenic	c.7055-1G>C g.73558867G>C	Pathogenicity score: 6; ACMG: Pathogenic
3 (∼2 years-old) (with CI)	90/110 Profound	c.995C>A g.73377011C>A (p.Thr332Lys)	Pathogenicity score: 14; ACMG: Pathogenic	c.5534A>G g.73544679A>G (p.Asn1845Ser)	Pathogenicity score: 14; ACMG: Likely Pathogenic
4 (2 years-old) (with CI)	100/100 Profound	c.2159G>A g.73450324G>A (p. Arg720Gln)	Pathogenicity score: 12; ACMG: Uncertain Significance	c.4762C>T g.73501595C>T (p.Arg1588Trp)	Pathogenicity score: 9; ACMG: Uncertain Significance

Notes: CI, cochlear implant. Five novel variants are in blod. Varsome: https://varsome.com/. ACMG: https://www.medschool.umaryland.edu/genetic_variant_interpretation_tool1.html/

### Genetic variants identified in half of examined patients

During the last 3 years, 351 individuals with congenital sensorineural deafness from 323 unrelated families were examined by exome sequencing. Among them, potential causal variants were identified in 166 affected individuals (51.4%). Among the 166 affected individuals, nearly one-third of them (54 individuals, 32.53%) were identified with variants within the *GJB2* gene. The second most frequently mutated gene in this cohort is *SLC26A4* (36 individuals, 21.69%). The third most frequently mutated gene in this cohort is the *MY O 15A* gene (15 individuals, 9.04%). Ten additional genes with 1%–5% mutation rates included *CHD7* (4.22%), *SOX10* (2.41%), *CDH23* (2.41%), *MYO7A* (1.81%), *MITF* (1.81%), *EYA1* (1.81%), *ESRRB* (1.20%), *PTPN11* (1.20%), *TECTA* (1.20%), and *PTPRQ* (1.20%).

### Identification of four compound mutations in *CDH23*

**FIGURE 2:**
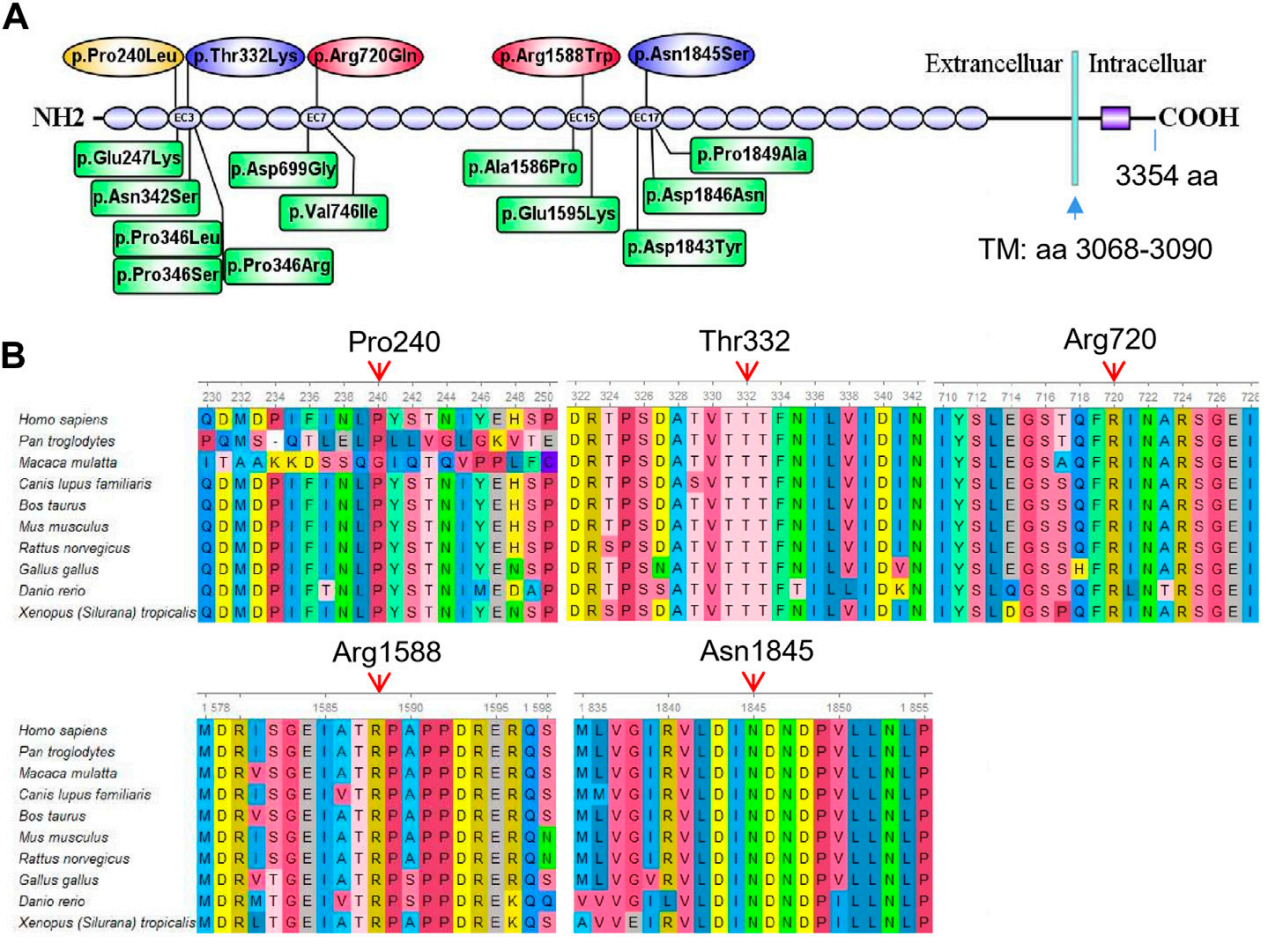
Mutation analysis of *CDH23*. **(A)** Five different missense mutations identified in the five affected individuals are mapped to the CDH23 protein. The spicing variant (c.7055-1G>C) in CDH23 is not shown in the CDH23 protein structure. As a comparison, 12 reported missense variants (HGMD) near the loci identified in the present study are also shown in green boxes. These missense variants are associated with nonsyndromic autosomal recessive deafness. Transmembrane domain (TM): amino acids 3,068–3,090. **(B)** All the five variants are evolutionarily conserved as shown in multiple sequence alignment containing 10 different species from Xenopus to Human.

In the present study, we report four different compound heterozygous mutations in the *CDH23* gene (NM_022124.6) from five patients in four families identified by exome sequencing (Table 1; Figure 2) and further confirmed by Sanger sequencing (Supplementary Figure S1). Among these mutations, only two variants (c.719C>T; p.Pro240Leu and c.4762C>T; p.Arg1588Trp) were previously reported (HGMD). Four variants in *CDH23* (Table 1) were not reported or curated in HGMD, including 1) c.7055-1G>C in family 2; 2) c.995C>A; p.Thr332Lys, and c.5534A>G; p.Asn1845Ser in family 3; 3) c.2159G>A; p.Arg720GLn in family 4. In addition, the affected child in family four also carried a heterozygous variant c.235delC (p.Leu79Cysfs*3) in *GJB2*, which was inherited from his phenotypically normal father. It is worthy to mention that homozygous c.235delC variants in *GJB2* were frequently detected in patients with recessive non-syndromic hearing loss due to a founder effect (Jiang et al., 2022).

### *In silico* analysis predicted pathogenic variants

Several major *in silico* variant prediction tools and molecular modeling were utilized to assess pathogenic effects of the detected variants on CDH23 protein structure, stability and function. The longest isoform of CDH23 (3,354 amino acids, GenBank acc. No. NP_071407.4) contains 26 extracellular cadherin (EC) repeats and one non-canonical domain (Figure 2A). Two missense variants (p.P240L and p.T332K) were gathered in the third EC repeat region (i.e., EC3; amino acids 258–346). The variant p.R720Q was in the seventh EC repeat (EC7, amin acids 693–776), p.R1588W in the 15th EC repeat (EC15, amino acids 1,550–1,632) and the p.N1845S in the 17th EC repeat (EC17, amino acids 1766–1849) (Figure 2A). Previously reported mutations in these four EC regions were linked to autosomal recessive nonsyndromic deafness and Usher syndrome 1D (Supplementary Table S2).

To examine the evolutionary conservation of the five amino acids that were mutated in the patients, CDH23 protein sequences of 10 different species from Xenopus to human were retrieved from reference sequences at NCBI (https://www.ncbi.nlm.nih.gov/gene/) for multiple sequence alignment analysis using Ugene program (http://ugene.net/). As shown in Figure 2B, all the five residues that were mutated in the affected individuals, including P240, T332, R720, R1588, and N1845, are evolutionarily conserved.

Furthermore, pathogenicities of these variants were predicted using several commonly used *in silico* tools. Both REVEL (Rare Exome Variant Ensemble Learner) and CADD (Combined Annotation Dependent Depletion) predicted that all the missense variants to be deleterious. More detailed analysis using Varsome online tool (Version 11.3) predicted all the variants (Table 1) to be pathogenic in quantitative scales (from pathogenicity score 6–14). Meanwhile, Varsome ACMG analysis predicted most of the variants (Table 1) to be pathogenic or likely pathogenic, except the last two variants (c.2159G>A and c.4762C>T) to be of uncertain significance.

**FIGURE 3:**
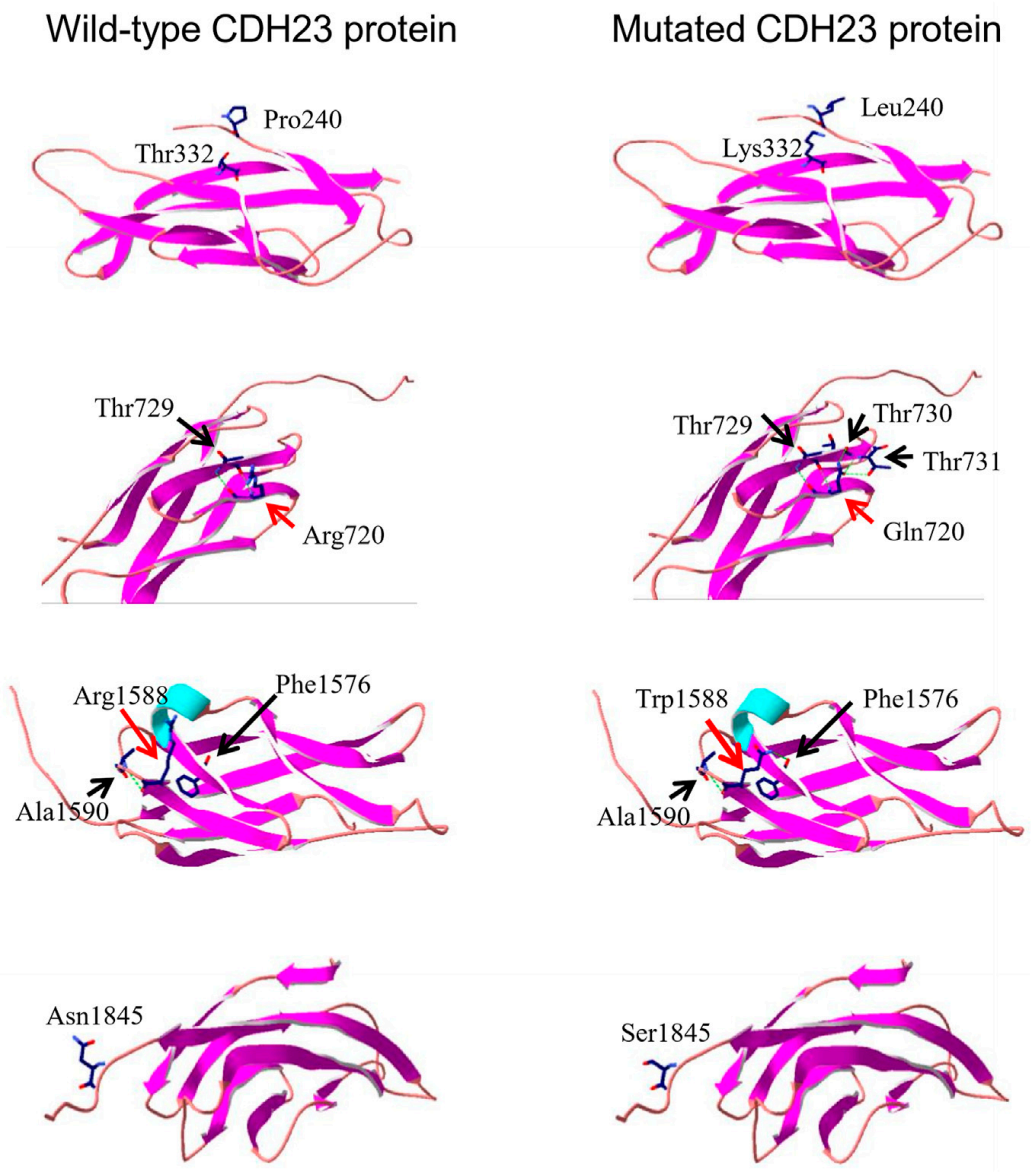
Three-dimensional protein structure analysis of five mutation-containing regions in CDH23. Four wild-type EC repeats (EC3, EC7, EC15, and EC17) CDH23 protein structures are listed on the left panels; four mutated counter parts are on the right. The locations of amino acid 240, 332, and 1845 do not have hydrogen bond formed with the surrounding amino acids. On the other hand, the residue R720 or R1588 may form several hydrogen bonds with surrounding amino acids (indicated by a dotted green line).

To look potential effects of the missense variants on CDH protein structure and associated functions, three-dimensional structures of four regions (EC3, EC7, EC15, and EC17, Figure 2A) of wild-type CDH23 were moldered using SWISS-MODEL (Figure 3, four panels on the left side). All the five missense variants in these regions may affect surrounding structure of CDH23 (Figure 3, four panels on the right side). Apparently, the wildtype Arg720 could form hydrogen bonds with Thr729. However, the Arg720Gln variant appeared to have five hydrogen bonds with three Thr residue from 729 to 731. Although other three variations were not found to affect hydrogen bond formation, the substitutions of charged residues are physiochemically significant.

## Discussion

In this study, we performed exome sequencing analysis for 351 affected individuals with hearing loss. Among the identified causal genes, variants in *GJB2* and *SLC26A4* were most frequently identified in this cohort, which is similar to previous reports (Fu et al., 2019; Xie et al., 2021). Here, we reported the identification of four novel variants in the *CDH23* gene (Table 1), which significantly expanded the mutation spectrum of *CDH23*-associated non-syndromic autosomal recessive deafness.

The *CDH23* gene is a member of the cadherin superfamily, which encodes a calcium-dependent cell–cell adhesion glycoprotein and is known to be expressed in inner and outer hair cells in the cochlea (Usami et al., 2022). Recessive mutations in *CDH23* can cause non-syndromic autosomal recessive deafness 12 (DFNB12, OMIM: 601386) and Usher syndrome type 1D deafness (USH1D, OMIM: 601067). DFNB12 is characterized by prelingual-onset sensorineural NSHL, without the impairment of visual or vestibular functions (Ramzan et al., 2020; Usami et al., 2022). On the other hand, USH1D (OMIM: 601067) is the most severe subtype and is characterized by a severe to profound prelingual SNHL, early retinitis pigmentosa onset and vestibular alterations (Fuster-Garcia et al., 2021).

To date, at least 492 different variants of *CDH23* have been curated in Human Gene Mutation Database (HGMD). Among them, missense/nonsense variants account for the majority, which are located in the extracellular region (Figure 2A). Missense variants in *CDH23* usually underlie a milder phenotype of NSHL. In contrast, protein-truncating-related *CDH23* mutations due to frameshift, splice site, or nonsense pathogenic variants are causative of the Usher syndrome with more severe phenotypes (Ramzan et al., 2020). However, compound heterozygous variants, if only one of the variants is a LoF allele, may not cause Usher syndrome like the affected child in family 2. In all detected CDH23 variants, p.Pro240Leu and p.Arg1588Trp are the most common variants in the Japanese hearing loss population (Usami et al., 2022). A meta-analysis indicated the p.Pro240Leu variant increased the risk of NHSL in Asian populations (Xu et al., 2019).

Notably, cadherin-23 is colocalized with protocadherin-15 (encoded by the *PCDH15* gene) in the hair-cell tip link (Sotomayor et al., 2010), a fine filament directly conveying force to mechanosensitive transduction channels. Mutations of *PCDH15* also cause similar disorders, including autosomal recessive deafness 23 (OMIM: 609533) and Usher syndrome type 1D (OMIM: 601067) or Usher syndrome type 1F (OMIM: 602083) (Ahmed et al., 2003; Doucette et al., 2009).

Lastly, affected individuals with hearing loss could benefit from the identification of their genetic causes by genomic sequencing, which may provide better preoperative evaluation and postoperative effect prediction for clinic and cochlear implant (CI). In fact, patients with *CDH23* mutations were predicted to acquire an acceptable auditory and speech outcome after CI (Liu et al., 2008; Yoshimura et al., 2020; Chen et al., 2022). In our study, two affected children in family three and family four underwent bilateral CI surgery at age of 2 years and indeed showed acceptable recovery.

## Conclusion

Taken together, we screened 351 affected individuals with hearing loss and their parents using exome sequencing. Here, we reported the identification of four novel variants (c.995C>A, p.T332K; c.2159G>A, p.R720Q; c.5534A>G, p.N1845S, and c.7055-1G>C) in the *CDH23* gene from five affected individuals with autosomal recessive non-syndromic deafness (OMIM: DFNB12). Our findings significantly expanded the mutation spectrum of *CDH23*-associated autosomal recessive hearing loss.

## Data Availability

The raw data supporting the conclusion of this article will be made available by the authors, without undue reservation.
