# Supplementary material for: Identification of four novel variants in the CDH23 gene from four affected families with hearing loss
Source: Front Genet. 2022 Nov 17;13:1027396. doi: 10.3389/fgene.2022.1027396 (PMC9713811; doi:10.3389/fgene.2022.1027396)
Supplement: Supplementary file 1 [file Table1.DOCX]

**Supplementary Table 1. Primers used for mutation analysis of the *CDH23* gene**

| Mutations | Forward primers | Reverse primers |
| --- | --- | --- |
| c.719C>T | CCTGCTTTGTAACACTGGATA | TGCCCTGGAAGCCTTTGTT |
| c.7055-1G>C | CCTCGGCAGGTAGGTTAG | CGATTTCCAGGTAGACAGG |
| c.2159G>A | GAGTCAGGATTTCACGG | TTCACCCTTGAAAGACG |
| c.4762C>T | ACCGTGACATCGGGATC | GCAGTGGTGCTGTTCATT |
| c.995C>A | CAGAACTGGGAATGGGCACT | TCCAGGGACTCACCTCAT |
| c.5534A>G | CATGCACAACATCTGTCGCT | TGAGAACTTTGGTGCGTCTT |
