# Supplementary figures and images for: Identification of four novel variants in the CDH23 gene from four affected families with hearing loss
Source: Front Genet. 2022 Nov 17;13:1027396. doi: 10.3389/fgene.2022.1027396 (PMC9713811; doi:10.3389/fgene.2022.1027396)

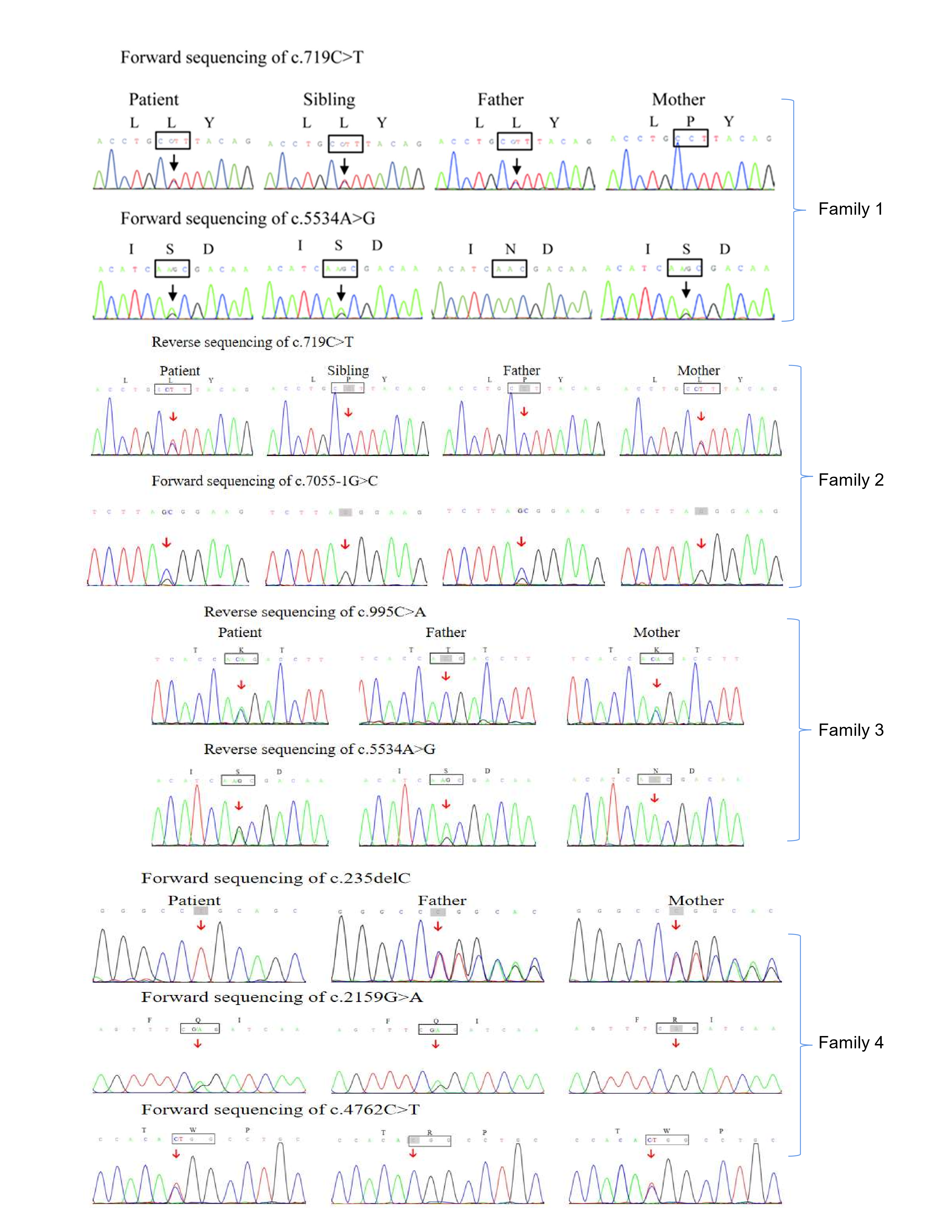

Supplement: Supplementary file 2 [file Image1.TIF]
