# Supplementary material for: Identification of four novel variants in the CDH23 gene from four affected families with hearing loss
Source: Front Genet. 2022 Nov 17;13:1027396. doi: 10.3389/fgene.2022.1027396 (PMC9713811; doi:10.3389/fgene.2022.1027396)
Supplement: Supplementary file 3 [file Table2.DOCX]

**Supplementary Table 2. Selected *CDH23* Variants and Associated Disorders**

| **Nucleotide change** | **Amino acid**  **change** | **Domain** | **Disease** | **References** |
| --- | --- | --- | --- | --- |
| c.739G>A | p.Glu247Lys;  p.Glu2554X | EC3;  LoF | **Usher syndrome 1D** | (Roux et al., 2006) |
| c.1025A>G | p.Asn342Ser | EC3 | Autosomal recessive nonsyndromic hearing loss | (Woo et al., 2014) |
| c.1037C>G | p.Pro346Arg | EC3 | hearing loss | (Sun et al., 2019) |
| c.2096A>G | p.Asp699Gly | EC7 | Autosomal recessive Sensorineural hearing impairment | (Zazo Seco et al., 2017) |
| c.4756G>C | p.Ala1586Pro | EC15 | Autosomal recessive nonsyndromic deafness | (Astuto et al., 2002) |
| c.4783G>A | p.Elu1595Lys | EC15 | Autosomal recessive nonsyndromic deafness | (Astuto et al., 2002) |
| c.5527G>T | p.Asp1843Tyr | EC17 | Autosomal recessive nonsyndromic hearing loss | (De Keulenaer et al., 2012) |
| c.5536G>A | p.Asp1846Asn | EC17 | Autosomal recessive nonsyndromic deafness | (Astuto et al., 2002) |
| c.5545C>G | p.Pro1849Ala | EC17 | Autosomal recessive nonsyndromic hearing loss | (Atik et al., 2015) |

**References**

Astuto, L.M., Bork, J.M., Weston, M.D., Askew, J.W., Fields, R.R., Orten, D.J., Ohliger, S.J., Riazuddin, S., Morell, R.J., Khan, S., Riazuddin, S., Kremer, H., Van Hauwe, P., Moller, C.G., Cremers, C.W., Ayuso, C., Heckenlively, J.R., Rohrschneider, K., Spandau, U., Greenberg, J., Ramesar, R., Reardon, W., Bitoun, P., Millan, J., Legge, R., Friedman, T.B., and Kimberling, W.J. (2002). CDH23 mutation and phenotype heterogeneity: a profile of 107 diverse families with Usher syndrome and nonsyndromic deafness. *Am J Hum Genet* 71**,** 262-275.

Atik, T., Onay, H., Aykut, A., Bademci, G., Kirazli, T., Tekin, M., and Ozkinay, F. (2015). Comprehensive Analysis of Deafness Genes in Families with Autosomal Recessive Nonsyndromic Hearing Loss. *PLoS One* 10**,** e0142154.

De Keulenaer, S., Hellemans, J., Lefever, S., Renard, J.P., De Schrijver, J., Van De Voorde, H., Tabatabaiefar, M.A., Van Nieuwerburgh, F., Flamez, D., Pattyn, F., Scharlaken, B., Deforce, D., Bekaert, S., Van Criekinge, W., Vandesompele, J., Van Camp, G., and Coucke, P. (2012). Molecular diagnostics for congenital hearing loss including 15 deafness genes using a next generation sequencing platform. *BMC Med Genomics* 5**,** 17.

Roux, A.F., Faugere, V., Le Guedard, S., Pallares-Ruiz, N., Vielle, A., Chambert, S., Marlin, S., Hamel, C., Gilbert, B., Malcolm, S., Claustres, M., and French Usher Syndrome, C. (2006). Survey of the frequency of USH1 gene mutations in a cohort of Usher patients shows the importance of cadherin 23 and protocadherin 15 genes and establishes a detection rate of above 90%. *J Med Genet* 43**,** 763-768.

Sun, Y., Xiang, J., Liu, Y., Chen, S., Yu, J., Peng, J., Liu, Z., Chen, L., Sun, J., Yang, Y., Yang, Y., Zhou, Y., and Peng, Z. (2019). Increased diagnostic yield by reanalysis of data from a hearing loss gene panel. *BMC Med Genomics* 12**,** 76.

Woo, H.M., Park, H.J., Park, M.H., Kim, B.Y., Shin, J.W., Yoo, W.G., and Koo, S.K. (2014). Identification of CDH23 mutations in Korean families with hearing loss by whole-exome sequencing. *BMC Med Genet* 15**,** 46.

Zazo Seco, C., Wesdorp, M., Feenstra, I., Pfundt, R., Hehir-Kwa, J.Y., Lelieveld, S.H., Castelein, S., Gilissen, C., De Wijs, I.J., Admiraal, R.J., Pennings, R.J., Kunst, H.P., Van De Kamp, J.M., Tamminga, S., Houweling, A.C., Plomp, A.S., Maas, S.M., De Koning Gans, P.A., Kant, S.G., De Geus, C.M., Frints, S.G., Vanhoutte, E.K., Van Dooren, M.F., Van Den Boogaard, M.H., Scheffer, H., Nelen, M., Kremer, H., Hoefsloot, L., Schraders, M., and Yntema, H.G. (2017). The diagnostic yield of whole-exome sequencing targeting a gene panel for hearing impairment in The Netherlands. *Eur J Hum Genet* 25**,** 308-314.
